# Alpha-Lipoic Acid and Leucine as Adjunct Therapy for Persistent First-Trimester Subchorionic Hematomas in Assisted Reproductive Technology Pregnancies

**DOI:** 10.7759/cureus.104107

**Published:** 2026-02-23

**Authors:** Tasneem Bano, Kedar Ganla, Priyanka H Vora

**Affiliations:** 1 Research and Development, Bellafem, Thane, IND; 2 Reproductive Medicine, Ankoor Fertility Clinic, Mumbai, IND

**Keywords:** alpha lipoic acid, assisted reproductive technologies, case series, first-trimester pregnancy, subchorionic hematoma

## Abstract

Subchorionic hematoma (SCH) is a frequent cause of first-trimester bleeding, particularly in pregnancies conceived through assisted reproductive technologies (ART), and is associated with adverse pregnancy outcomes when persistent. Management is largely conservative, with limited targeted therapeutic options. This descriptive case series evaluated the effectiveness and safety of oral alpha-lipoic acid (ALA) as an adjunct therapy in three primigravida women with ART-conceived pregnancies complicated by persistent first-trimester SCH unresponsive to conventional management, including progesterone support and hemostatic agents. ALA was administered orally at a dose of 600 mg once daily, alongside continued standard therapy. Clinical symptoms and hematoma progression were monitored through serial transvaginal ultrasonography. All three patients experienced cessation of vaginal bleeding within four to seven days of initiating ALA therapy. Complete sonographic resolution of the hematoma was observed within two to four weeks in all cases. No maternal or fetal adverse effects were reported, and all pregnancies progressed uneventfully into the second trimester. These findings suggest that oral ALA may represent a safe and potentially effective adjunctive therapy for persistent first-trimester SCH in ART pregnancies, warranting further evaluation in larger prospective studies.

## Introduction

First-trimester bleeding affects approximately 16-25% of all pregnancies and remains one of the most frequent causes of early obstetric consultation [1]. Among the various etiologies, subchorionic hematoma (SCH) is the most commonly identified ultrasonographic finding and is reported in 1-22% of pregnancies depending on the population studied and the timing of imaging [2]. SCH is defined as the accumulation of blood between the chorionic membrane and the uterine decidua, resulting from partial separation of the chorion from the uterine wall.

The clinical significance of SCH is heterogeneous and depends on factors such as hematoma size, location, placental involvement, and gestational age at diagnosis [2-4]. Several systematic reviews and observational studies have reported an association between SCH and adverse pregnancy outcomes, including early pregnancy loss, placental abruption, fetal growth restriction, preterm birth, and premature rupture of membranes, particularly when hematomas are large or persistent [2,4,5]. However, findings across studies remain inconsistent, and the prognostic implications of SCH continue to be debated.

The pathophysiology of SCH is thought to involve defective trophoblastic invasion, decidual vascular fragility, impaired uteroplacental perfusion, and dysregulated local hemostasis at the maternal-fetal interface. Oxidative stress has also been implicated in abnormal placentation and early gestational bleeding, particularly in high-risk pregnancies [6].

Pregnancies conceived through assisted reproductive technologies (ART) demonstrate a higher incidence of SCH compared with spontaneous conceptions [6]. Altered implantation dynamics, supraphysiological hormonal exposure, and impaired endometrial receptivity in ART cycles may predispose to abnormal placentation and early decidual hemorrhage.

Alpha-lipoic acid (ALA) is an endogenous antioxidant and mitochondrial cofactor with free-radical scavenging, anti-inflammatory, and endothelial-stabilizing properties. ALA has been shown to improve microvascular function and support placental health in conditions associated with oxidative stress [7,8]. Given the absence of targeted therapies for persistent SCH, this case series aims to describe the clinical and sonographic outcomes of adjunctive oral ALA therapy in ART-conceived pregnancies complicated by first-trimester SCH.

## Case presentation

Case 1

**Figure 1:**
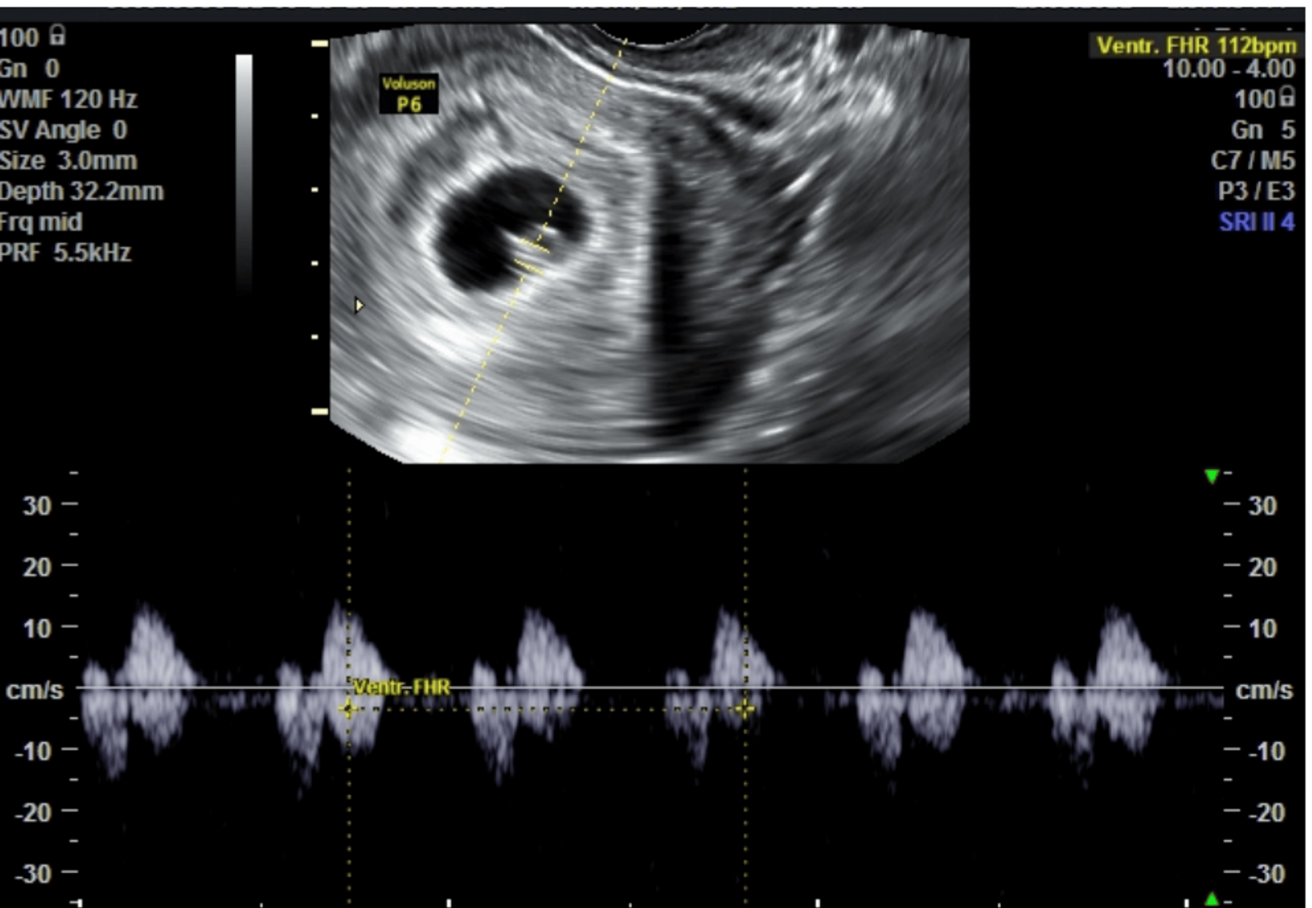
Transvaginal ultrasound (Case 1, 6.5 weeks) demonstrating a 2.3 × 1.7 cm subchorionic hematoma adjacent to a live intrauterine gestation.

**Figure 2:**
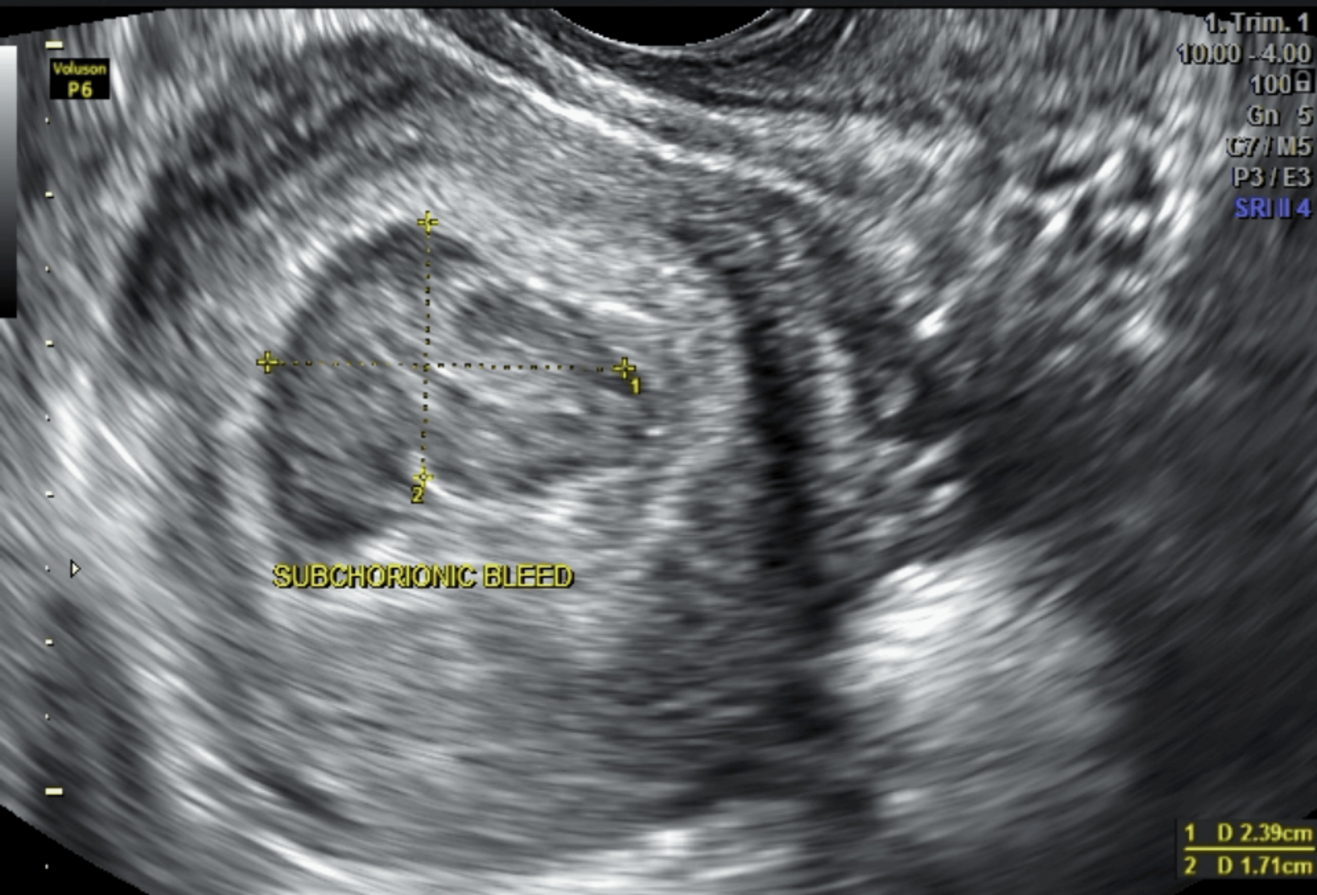
Magnified view of the 2.3 × 1.7 cm hematoma in Case 1, showing its crescent-shaped hypoechoic appearance beneath the chorionic membrane.

A 36-year-old primigravida with a six-year history of primary infertility conceived via in vitro fertilization and embryo transfer. She had no history of hypertension, diabetes, thyroid disease, thrombophilia, smoking, or prior uterine surgery. Her BMI was 23 kg/m². She presented at 6.5 weeks with mild spotting. Transvaginal ultrasound showed a viable intrauterine gestation and a posterior SCH measuring 2.3 × 1.7 cm (Figures 1, 2).

**Figure 3:**
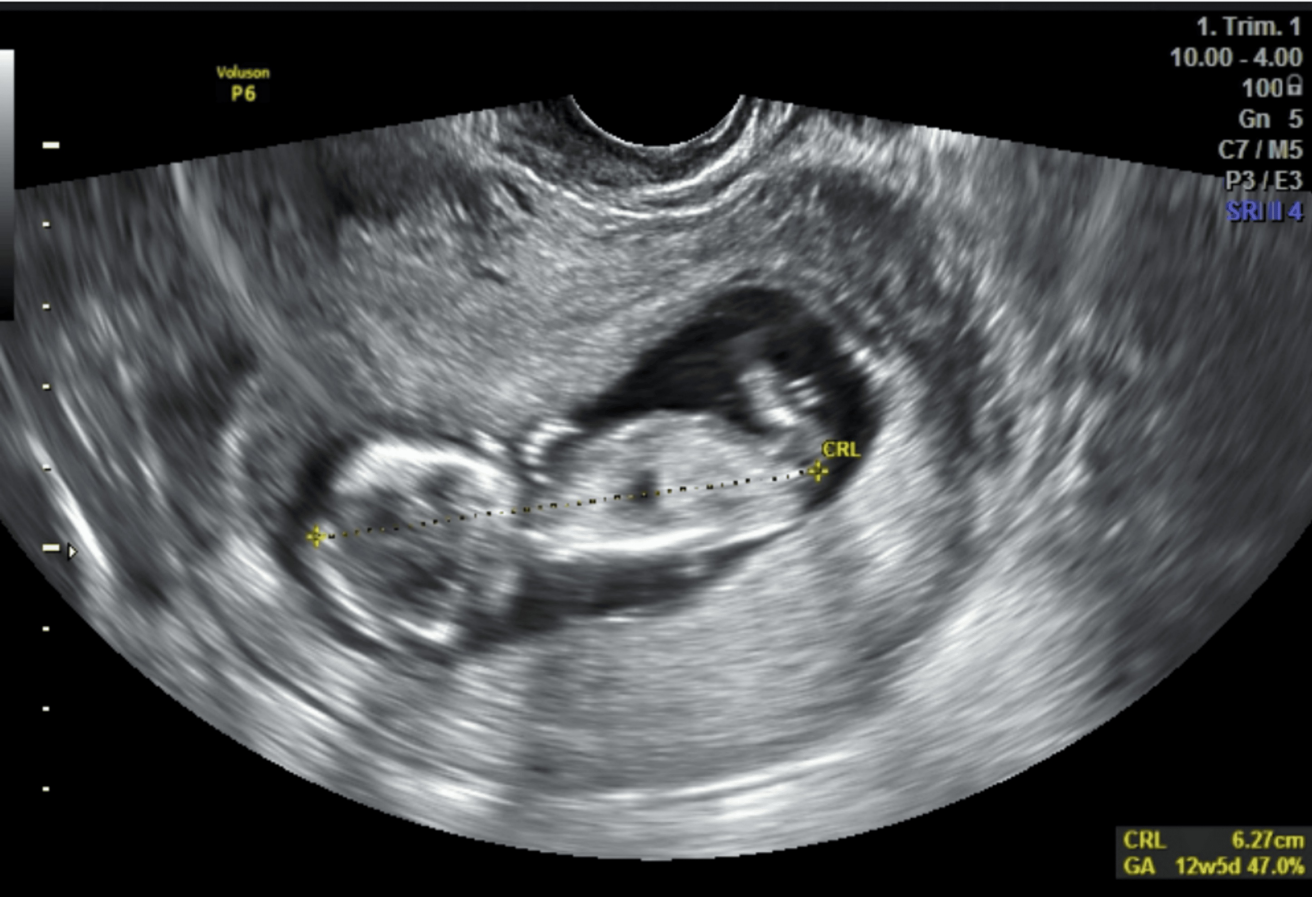
Follow-up ultrasound in Case 1 after initiating oral alpha-lipoic acid, illustrating complete resorption of the hematoma.

Initial management consisted of progesterone support and oral tranexamic acid. Due to persistence for 14 days, oral ALA 600 mg once daily was initiated in addition to ongoing conservative therapy. Follow-up imaging demonstrated progressive reduction and complete resolution (Figure 3).

Case 2

**Figure 4:**
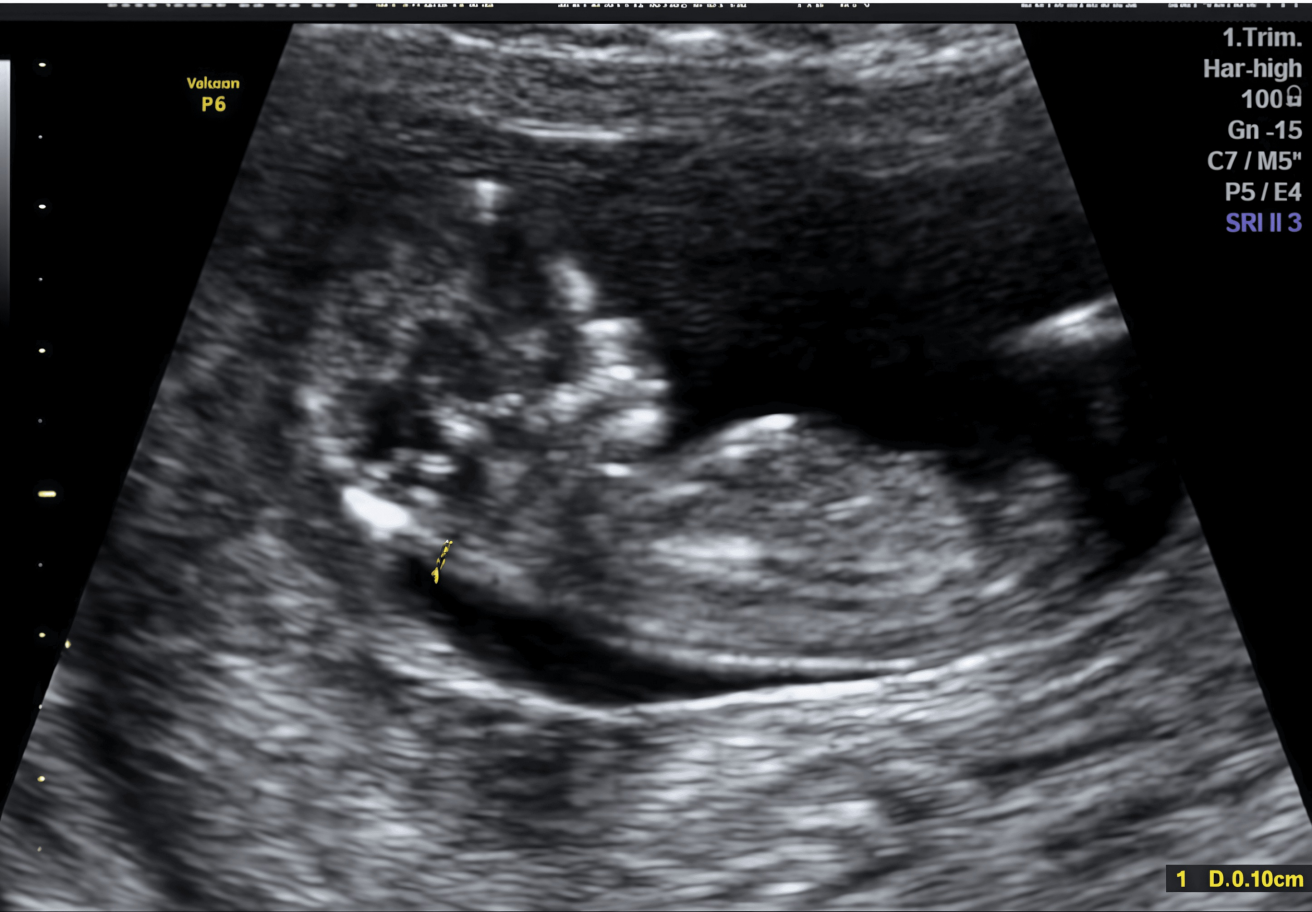
Transvaginal scan (Case 2, six weeks) revealed a large 3.1 × 2.66 cm subchorionic hematoma despite initial conservative therapy.

A 32-year-old primigravida with tubal factor infertility conceived via frozen embryo transfer. No medical comorbidities, coagulation disorders, or prior pregnancy losses were reported. Her BMI was 24.1 kg/m². She presented at seven weeks with moderate bleeding (1-2 pads/day). Ultrasound demonstrated a viable embryo and anterior SCH measuring 3.1 × 2.66 cm despite initial therapy (Figure 4).

**Figure 5:**
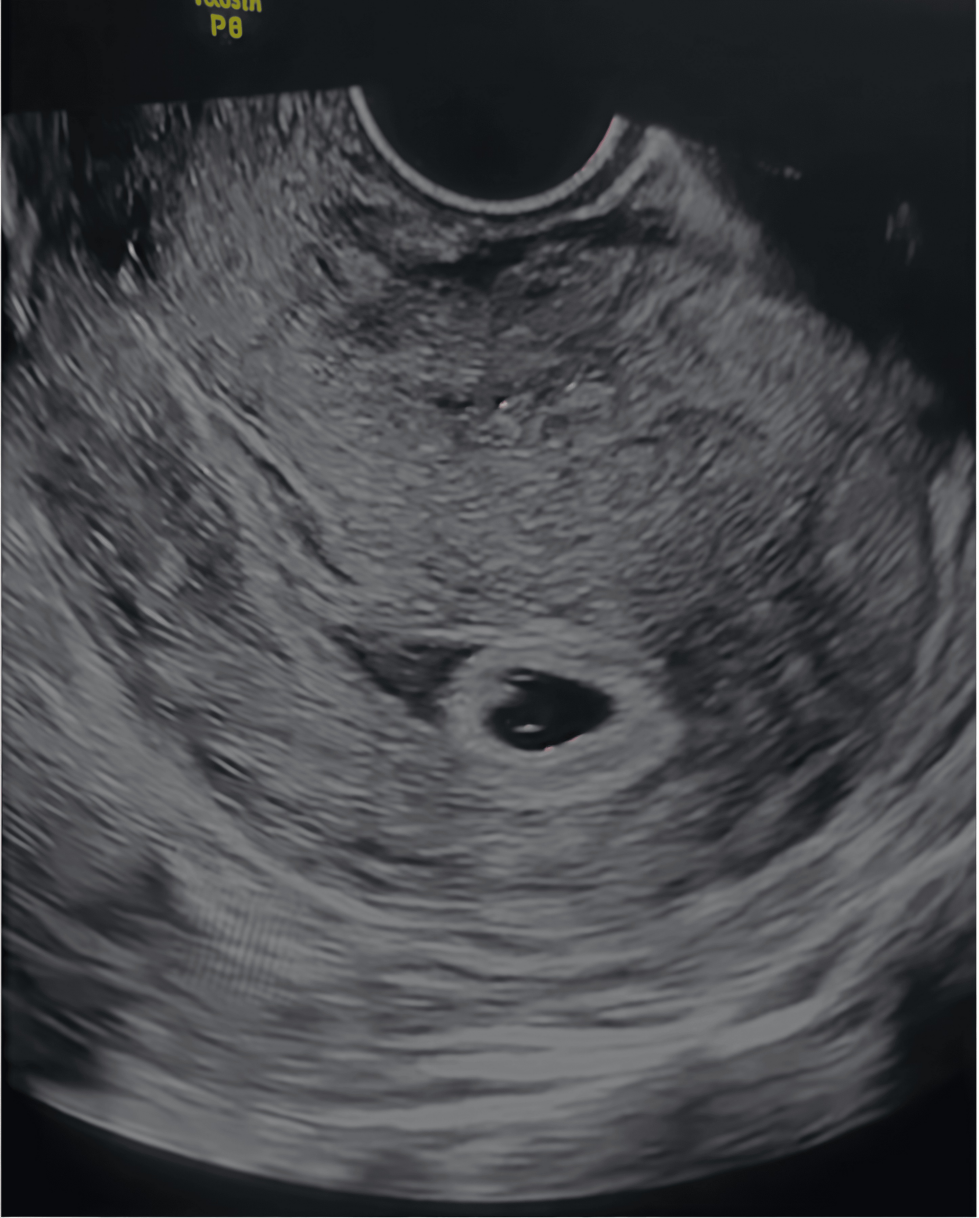
Follow-up scan in Case 2 at 12 weeks showing full resolution of the hematoma following adjunctive alpha-lipoic acid treatment.

Progesterone and tranexamic acid were started initially. After 10 days without improvement, oral ALA 600 mg daily was added alongside conservative therapy. Subsequent imaging showed marked reduction, followed by complete resolution by 12 weeks of gestation (Figure 5).

Case 3

A 34-year-old primigravida with diminished ovarian reserve conceived through intrauterine insemination. She had no systemic illness, no uterine anomalies, and no history of anticoagulant use or smoking. Her BMI was 22.8 kg/m². She presented at 7.5 weeks with mild spotting without clots. Ultrasound revealed a viable pregnancy with an inferior SCH measuring 2.5 × 2.75 cm (Video 1). After 10 days of progesterone and tranexamic acid therapy failure, oral ALA 600 mg daily was introduced as an adjunct treatment. Serial ultrasonography demonstrated progressive shrinkage and eventual complete resolution of the hematoma (Video 1).

**Video 1:** This video demonstrates serial transvaginal ultrasonography of a patient diagnosed with a subchorionic hematoma in early pregnancy. The initial scan shows a crescent-shaped hypoechoic area adjacent to the gestational sac, consistent with a subchorionic hematoma. Subsequent frames illustrate progressive reduction and eventual complete resolution of the hematoma following oral alpha-lipoic acid therapy, with preservation of a viable intrauterine gestation.

Results

Serial ultrasonography demonstrated a reduction in hematoma dimensions from 2.3 × 1.7 cm to complete resolution (Case 1), 3.1 × 2.66 cm to undetectable (Case 2), and 2.5 × 2.75 cm to complete resolution (Case 3). All patients were followed until completion of the first trimester (range: 12-13 weeks of gestation), during which no recurrence of bleeding or hematoma was observed.

Bleeding severity at presentation was classified clinically as follows: case 1: mild spotting (pantyliner level); case 2: moderate bleeding (1-2 pads/day); case 3: mild spotting without clots.

Summary of cases

Clinical characteristics and treatment outcomes of all three patients are summarized in Table 1.

**Table 1:** Summary of case findings. ART: assisted reproductive technology; ICSI: intracytoplasmic sperm injection; FET: frozen embryo transfer; IUI: intrauterine insemination; TXA: tranexamic acid; ALA: alpha-lipoic acid.

Case	Age	ART method	Hematoma size	Initial management	ALA response	Time to resolution
1	30	ICSI	2.3 × 1.7	Progesterone + TXA	Bleeding stopped in 7 days	2 weeks
2	35	ICSI + FET	3.1 × 2.66	Progesterone + TXA	Spotting ceased in 4 days	4 weeks
3	34	IUI	2.5 × 2.75	Progesterone + TXA	Bleeding stopped in 5 days	3 weeks

Baseline demographic and ultrasound parameters are presented in Table 2.

**Table 2:** Baseline characteristics. GA: gestational age; CRL: crown-rump length; FHR: fetal heart rate.

Variable	Case 1	Case 2	Case 3	Mean/range
Age	30	35	34	33 ± 2.6
GA at diagnosis	6.5 weeks	8 weeks	7.5 weeks	7.3 ± 0.76
CRL	9.2	15	11	11.7 ± 2.9
FHR	112	140	120	124 ± 14

Symptom evolution before and after ALA initiation is shown in Table 3.

**Table 3:** Symptom status.

Symptom	Case 1	Case 2	Case 3
Bleeding at presentation	Yes	Yes	Yes
Pain	No	No	No
Bleeding stopped after alpha-lipoic acid	Yes	Yes	Yes
Recurrence	No	No	No
Maternal side effects	None	None	None

All patients were followed until completion of the first trimester (12-13 weeks), with no recurrence of bleeding.

Quantitative treatment outcomes and resolution timelines are summarized in Table 4.

**Table 4:** Clinical and ultrasonographic outcomes.

Parameter	Case 1	Case 2	Case 3	Mean
Bleeding cessation	7 days	4 days	5 days	5.3 days
Time to resolution	2 weeks	4 weeks	3 weeks	3 weeks
Reduction at 2 weeks	100%	42%	70%	71%
Gestational age at resolution	8.5 weeks	12 weeks	10.5 weeks	-

## Discussion

The present case series demonstrates a consistent temporal association between initiation of ALA and rapid clinical improvement across all three patients with persistent SCH unresponsive to standard therapy. Notably, bleeding cessation occurred within one week in each case, followed by progressive sonographic reduction and eventual hematoma resolution. Persistent SCH has previously been associated with increased risk of adverse pregnancy outcomes, particularly when large or prolonged in duration [2,4,5].

The uniform response pattern observed across patients with different ART modalities suggests that the therapeutic effect may be independent of conception technique and instead related to shared underlying mechanisms such as oxidative stress-mediated endothelial dysfunction. Oxidative stress has been implicated as a key contributor to abnormal placentation and early gestational bleeding, particularly in ART pregnancies [6]. The rapidity of symptom resolution following ALA introduction, despite prior failure of conventional therapy, strengthens the hypothesis of a pharmacologic contribution rather than spontaneous resolution alone.

Known demographic and clinical factors associated with increased SCH risk include advanced maternal age, infertility treatment, prior miscarriage, uterine structural abnormalities, and coagulation disorders [2,5]. None of these major systemic risk factors were present in our patients except ART conception, suggesting that the observed response was unlikely to be attributable to correction of underlying systemic pathology.

Another clinically relevant observation is that all hematomas resolved before the end of the first trimester, a time frame associated with improved pregnancy prognosis in prior observational studies [2,4]. This suggests that early adjunctive antioxidant therapy may shorten hematoma persistence duration. ALA has demonstrated antioxidant, endothelial-stabilizing, and placental-protective properties in both clinical and preclinical studies [7,8].

While spontaneous resolution remains possible, the consistent improvement shortly after ALA initiation across multiple cases indicates a signal that merits prospective validation.

A limitation of this series is that patients were receiving concomitant antifibrinolytic and progesterone therapy prior to ALA initiation. Although these therapies had failed to produce clinical improvement before ALA was introduced, their potential synergistic contribution to clot stabilization cannot be fully excluded. Controlled trials evaluating ALA monotherapy versus combination therapy are warranted.

## Conclusions

Adjunctive oral ALA therapy was associated with rapid clinical improvement and complete sonographic resolution of persistent first-trimester SCHs in ART-conceived pregnancies. These findings should be interpreted as hypothesis-generating and should not be considered evidence of efficacy until validated in controlled trials.
